# Ni-Doped SnO Microplates for Carbon Monoxide Gas Detection

**DOI:** 10.1021/acsomega.5c06392

**Published:** 2025-10-07

**Authors:** Giuliana Giulietti, Miguel D. Sanchez, Elson Longo, Marcelo Assis, Anderson Albuquerque, Julio R. Sambrano, Miguel A. Ponce, Paula M. Desimone

**Affiliations:** a Institute for Research in Materials Science and Technology (INTEMA), 28233 National University of Mar del Plata (UNMdP), Mar del Plata B7600, Argentina; b Instituto de Física del Sur (IFISUR), Departamento de Física, Universidad Nacional del Sur (UNS), CONICET, Bahía Blanca 8000, Argentina; c Center for Research and Development of Functional Materials (CDMF), 67828 Federal University of São Carlos (UFSCar), São Carlos, SP 13565, Brazil; d Department of Biosciences, Federal University of São Paulo (UNIFESP), Santos 11015-020, Brazil; e Instituto de Química, Universidade Federal do Rio Grande do Norte, UFRN, Natal, RN 59078, Brazil; f Modeling and Molecular Simulations Group, São Paulo State University (UNESP), School of Sciences, Bauru, SP 17033, Brazil; g CIFICEN (UNCPBA-CICPBA-CONICET) and Instituto de Física de Materiales Tandil (UNCPBA), Tandil B7000, Argentina

## Abstract

Undoped and Ni-doped SnO were synthesized using a microwave-assisted hydrothermal method to analyze the influence of Ni-doped SnO nanostructures on CO detection. The scanning electron microscopy (SEM) analysis revealed a predominantly tetragonal SnO phase, with a minor proportion of the tetragonal SnO_2_ phase. X-ray photoelectron spectroscopy (XPS) confirmed the SnO phase without the NiO_*x*_ phase on the microplate surfaces. The images showed micrometric plates with SnO (001) surfaces. The presence of Ni led to an increase in the carrier concentrations, resulting in enhanced conductivity. Additionally, density functional theory (DFT) calculations indicated that Ni doping in the outermost layer significantly enhanced CO affinity via carbon coordination, while oxygen-bound configurations became unstable. Electrical measurements showed a slight decrease in the activation energy (*E*_a_) for the Ni-doped sample under a reductive atmosphere. This behavior facilitates the use of this material at room temperatures, which is technologically desirable for CO sensor devices.

## Graphical abstract

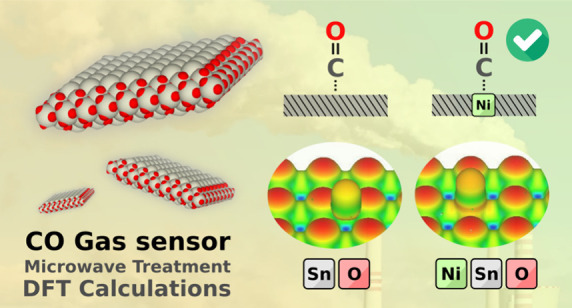

## 1 Introduction

Several factors, including the n- or p-type semiconductor, vacancy concentration, and nanoparticle morphology, can significantly influence sensor performance.^1,2^ Among these, the doping strategy has emerged as a strategy to enhance sensing characteristics. By incorporating transition metals as dopants, the electronic and chemical structures of semiconductors can be effectively modified, directly impacting their sensitivity and selectivity toward several gases.^1^

Moreover, it is well established that improving sensor precision and durability requires the development of more sensitive and stable sensing materials.^3–5^

SnO, in particular, still retains the significant interest of the scientific community as a material with immense potential for various technological applications.^6^ With a band gap energy (*E*_g_) ranging from 2.5 to 3.0 eV,^7–9^ SnO demonstrates technological potential in critical fields such as gas sensing, photocatalysis, and transparent conductive electronics.^10,11^ Within the realm of solid-state gas sensors, tin oxide stands as a noteworthy contender, taking the form of two distinct oxides: tin­(II) oxide (SnO), as a p-type semiconductor, and tin­(IV) oxide (SnO_2_), as an n-type semiconductor.

Two primary strategies exist to modify the SnO band gap energy. The first approach involves dimensional reduction, exemplified by SnO nanosheets, which typically exhibit a wider *E*_g_ compared to bulk SnO.^12,13^ The second approach employs the introduction of impurities or dopants into the SnO, thereby increasing carrier concentration and subsequently modifying the *E*_g_ *,* culminating in improved sensor responses.^9,14^ This strategy creates fertile ground for the development of more efficient and adaptable nanoparticles that can be used as gas sensors to detect atmospheric pollutants.

Particularly, nickel was usually used to dope n-type semiconductors like ZnO and SnO_2_ in order to improve their sensing properties.^15–17^ However, to the best of our knowledge, there are no studies dedicated to understanding the electrical conduction mechanisms in films formed with micrometric plates of Ni-doped SnO.

Considering this context, this paper presents the synthesis of pure and 1% Ni-doped SnO through a microwave-assisted hydrothermal (MAH) method and, subsequently, assesses their performance for carbon monoxide (CO) detection. These samples have been subjected to a comprehensive analysis involving various techniques such as X-ray diffraction (XRD), Raman spectroscopy, diffuse reflectance spectroscopy (DRS), X-ray photoelectron spectroscopy (XPS), scanning/transmission electron microscopy (SEM/TEM), and density functional theory (DFT) simulations.

This research also delves into an in-depth exploration of the structural and electronic attributes of the synthesized materials. The ultimate goal is to unravel the electronic conduction mechanisms governing this material, thereby not only advancing their comprehension but also underscoring their significant potential for practical applications in CO atmospheres.

## 2 Experimental Section

### 2.1 Synthesis Procedure

The SnO samples were synthesized by using the MAH method. Materials were tin­(II) chloride dihydrate (SnCl_2_·2H_2_O) (Synth, 98% purity), urea (Sigma-Aldrich, 98% purity), hydrochloric acid (Synth, 37%), and nickel­(II) nitrate hexahydrate (Ni­(NO_3_)_2_·6H_2_O) (Sigma-Aldrich, 98.5% purity). SnCl_2_·2H_2_O (5.76 mmol) was dissolved in 50 mL of distilled water under constant magnetic stirring at room temperature. After this process, 0.7 mL of hydrochloric acid was added, followed by the addition of 21.65 mmol of urea and 50 mL of distilled water. Once the solutions were homogenized, they were placed in a Teflon reactor and subjected to microwave treatment (2.45 GHz, with a maximum power of 800 W). The microwave operated at a temperature of 160 °C for 32 min. Finally, the samples were washed with distilled water five times using a centrifuge (5000 rpm/3 min) and dried for 12 h at 60 °C. For the sample doped with 1% Ni, all processes were similar, but 5.6446 mmol of SnCl_2_·2H_2_O was added, followed by the addition of 0.0576 mmol of Ni­(NO_3_)_2_·6H_2_O. A schematic representation of the synthesis route is shown in Figure 1.

**Figure 1:**
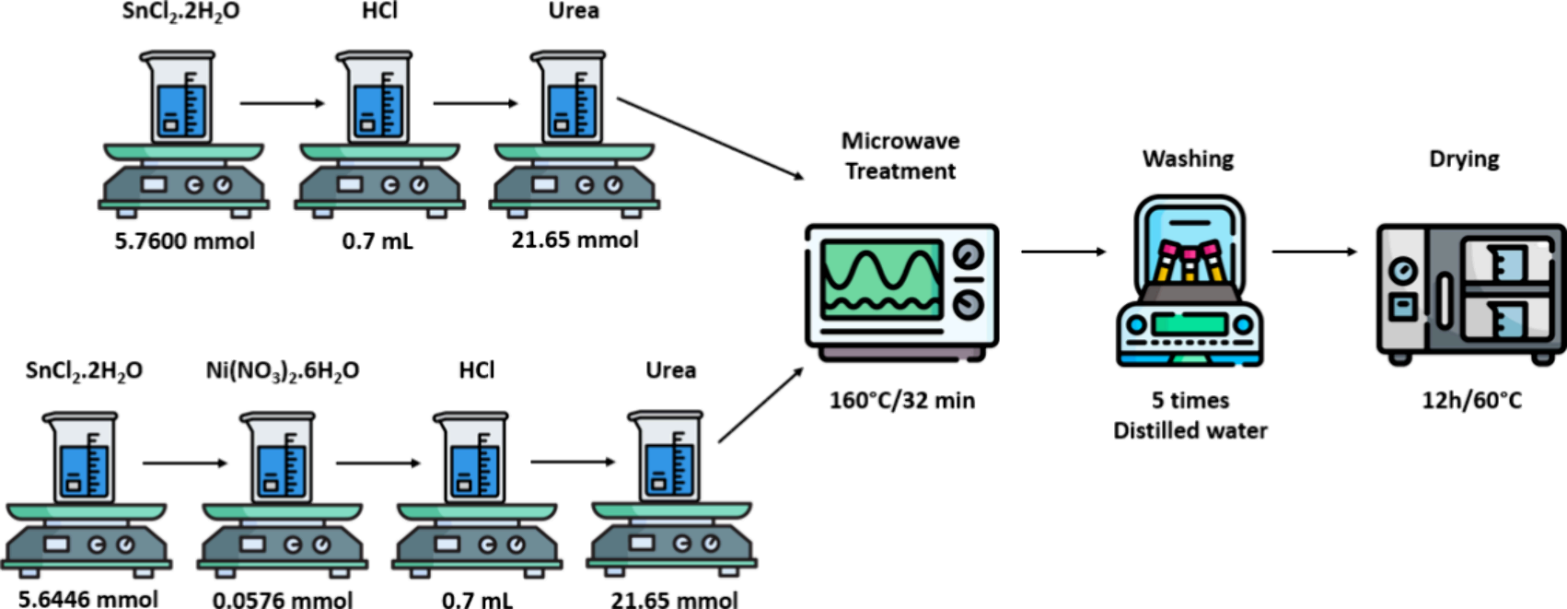
Schematic representation of the synthesis route.

### 2.2 Characterizations

The structural and optical properties of each sample were thoroughly examined using a selection of advanced techniques:

i) XRD analysis was used to characterize the crystalline phase, utilizing a D/Max-2500PC diffractometer (Rigaku, Japan) with Cu Kα radiation (λ = 1.5406 Å) in the 2θ range of 10–90° at a scan speed of 1° min^–1^.
ii) The Rietveld refinement method was applied within the GSAS I software^18^ with the objective of evaluating the crystal structures of the synthesized samples.
iii) Raman spectroscopy, with an iHR550 spectrometer equipped with a charge-coupled device detector and an argon-ion laser operating at 633 nm (with a power output of 200 mW), provided further insights.
iv) Optical properties were investigated via DRS using a Varian model 5G spectrometer.
v) Band gap energy inference was accomplished by transforming the UV–vis data into Tauc plots through the Kubelka–Munk function.^19^
vi) Morphological assessments were conducted using an SEM Supra 35-VP microscope at 5 kV, while TEM analysis was performed using a JEOL 2100 microscope equipped with an energy dispersive spectroscopy (EDS) system INCA Energy TEM 200, operating at 200 kV.
vii) XPS was performed using a PHI 548 spectrometer using the nonmonochromatic Al Kα radiation at 250W and 20 mA. The resolution spectra were taken at 50 eV pass energy, giving an energy resolution of ±0.5 eV. The operation base pressure was better than 5 × 10^–9^ Torr.
viii) The binding energy of the adventitious carbon was taken as an internal charge reference fixed at 284.8 eV. Signal fitting was made using Shirley-type background subtraction and the sum of Gaussian–Lorentzian functions as a peak model, and the surface atomic concentration estimations were done by relating the peak areas after the background subtraction and corrected relative to the corresponding atomic sensitivity.

### 2.3 Electrical Characterization

A paste composed of pristine and Ni-doped SnO powders, utilizing 1,2-propanediol as the organic binder, was meticulously prepared and deposited onto a silicon wafer acting as a substrate (pristine and Ni-doped SnO films). For the fabrication of an interdigitated structure, a 200 nm thick platinum layer was sputter-deposited onto the substrate, as detailed elsewhere.^20^ Following paste deposition and to enhance adhesion and facilitate binder evaporation, the samples were subjected to a 24 h drying process at 100 °C in ambient air. Subsequently, the samples underwent a heating step at 200 °C for 1 h in an air atmosphere, with a heating rate of 1 °C/min, aimed at completely evaporating the binder. After pristine and Ni-doped SnO film preparation, electrical measurements were conducted.

The investigation of the electrical behavior in the presence of carbon monoxide provides valuable insights into the response mechanisms. The measurements took place within a controlled chamber, ensuring the precise regulation of temperature, pressure, and gas composition. Electrical resistance was quantified by using an Agilent 3440A multimeter. Initially, three heating and cooling cycles were carried out in the presence of air (1 atm), vacuum (10^–4^ atm), and CO (6.6 × 10^–3^) atm to evaluate the performance of the films at specific temperatures within these distinct atmospheres. Subsequently, electrical resistance was monitored in air, vacuum, and CO (6.6 × 10^–3^and 6.6 6.6 × 10^–2^ atm) for 10 min in each atmosphere at 250 °C.

By tracking changes in electrical conductivity, it was feasible to ascertain the material response under different atmospheres. The measurement of response time (*t*_resp_) and recovery time (*t*_rec_) involves monitoring the duration required for the SnO material to react to the presence of a gas and return to its initial state once the gas was removed, respectively. These *t*_resp_ and *t*_rec_ values are indicative of the material dynamic behavior and hold significant relevance for real-time gas sensing applications.

### 2.4 Computational Methods

All electronic structure calculations were performed using spin-polarized density functional theory (DFT) within the Quantum ESPRESSO package (v7.1).^21^ We employed the PBE exchange-correlation functional^22^ with Grimme’s DFT-D3 dispersion corrections^23^ to account for van der Waals interactions. The calculations utilized ultrasoft Vanderbilt pseudopotentials (USPP),^24^ with kinetic energy cutoffs of 50 Ry for wave functions and 500 Ry for charge density (10× the wave function cutoff). Electronic convergence was achieved with thresholds of 10^–6^ Ry for total energy and 10^–5^ Ry/Bohr for forces. Structural optimizations were performed using the default BFGS algorithm with convergence criteria of 10^–4^ Ry/Bohr for atomic forces and 10^–3^ Bohr for atomic displacements. The Brillouin zone was sampled at the Γ-point for all systems using 3 × 3 × 3 and 3 × 3 × 1 Monkhorst–Pack k-point grids for bulk and surface (slab) models, respectively, which proved sufficient for these large supercells.

## 3 Results and Discussion

### 3.1 Characterizations

To study the structural and morphological characterization of the synthesized particles, diverse techniques were carried out for pure and Ni-doped SnO films.

Figure 2A presents the XRD analysis performed to identify the phase composition and degree of order/disorder in the long-range crystalline structure of the samples. Both XRD patterns exhibit distinct peaks, confirming the crystalline nature of the sample. For both pure and Ni-doped films, diffraction peaks corresponding to the tetragonal phase of SnO (ICSD 15516) are predominantly observed.^25^ However, for the pure SnO sample, some additional broadened peaks are visible at approximately 27, 34, and 52°, corresponding to tetragonal SnO_2_ (ICSD 9163), indicating that the 1% Ni doping decreases the formation of SnO_2_. Besides, no secondary phases such as NiO were detected, indicating that Ni is incorporated into the SnO matrix. Additionally, a slight shift of the (101) peak in the Ni-doped sample is observed compared to the pure sample at higher 2θ angles due to the smaller ionic radius of Ni^2+^ (0.55 Å) compared to Sn^2+^ (0.93 Å).^9^ This analysis confirms that Ni^2+^ ions substitute for Sn^2+^ cations in the lattice. An in-depth analysis of the composition and crystalline structure was conducted through Rietveld refinement analyses, as shown in Table 1. The reliability parameters (*R*_p_, *R*_wp_, and χ^2^) indicate high agreement between the calculated and observed XRD data. Furthermore, it is evident that, as previously observed, the pure SnO sample contains 8.0% SnO_2_ in its composition, while the 1% Ni-doped SnO sample contains only 2.3%, confirming the decrease of the SnO_2_ phase. Additionally, the shift observed in the diffractograms confirms a slight decrease in the unit cell volume of the Ni-doped sample.

**Figure 2:**
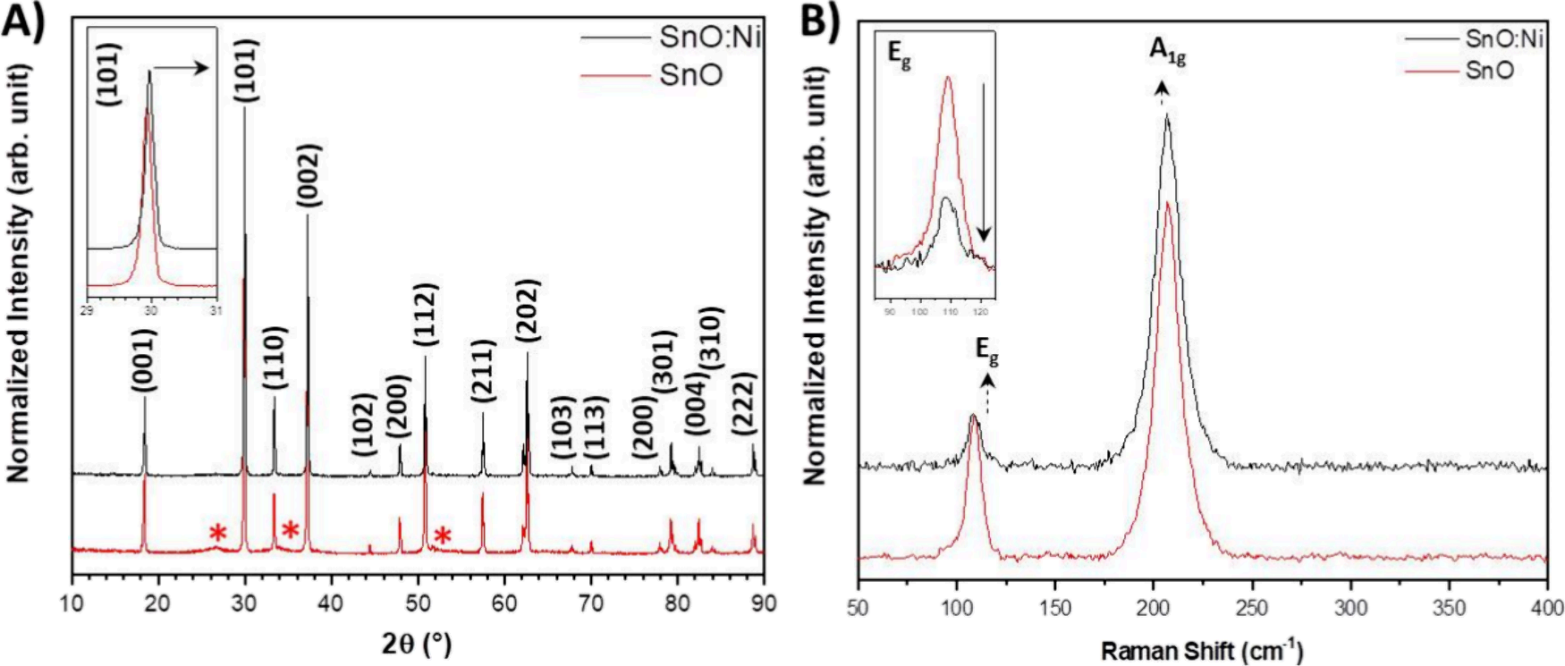
(A) XRD and (B) Raman spectra of the samples.

**Table 1:** Rietveld Analysis Parameters^a^

sample	ICSD	phase (%)	*a* (Å)	*c* (Å)	*V* (Å^3^)	*R*_p_ (%)	*R*_wp_ (%)	χ^2^
SnO	SnO	92.0	3.801(6)	4.837(2)	69.909	14.88	8.03	1.653
	SnO_2_	8.0	4.716(4)	3.159(8)	70.290			
SnO:Ni	SnO	97.7	3.801(9)	4.835(6)	69.896	12.64	7.66	1.542
	SnO_2_	2.3	4.727(4)	3.139(4)	70.162			

a *a* and *c* are lattice parameters that define the dimensions of the unit cell of a crystal. *V* is the volume of the unit cell, typically expressed in cubic angstroms (Å^3^). The unit cell volume is calculated directly from the refined lattice parameters (*a*, *b*, and *c* and the interaxial angles). *R*_p_, *R*_wp_, and χ^2^ (chi-squared) are critical parameters used to assess the goodness of fit between the experimentally observed X-ray (or neutron) diffraction pattern and the theoretically calculated pattern.

Raman spectroscopy is often employed for the analysis of short-range order in crystalline materials, serving as a complementary approach to XRD.^26^ In the case of the tetragonal SnO phase, the pure and doped SnO Raman spectra (Figure 2B) display vibrational modes at 109 cm^–^^1^ (E_g_ mode) and 207 cm^–^^1^ (A_1g_ mode), corresponding to its characteristic SnO tetragonal structure.^27,28^ The presence of these Raman modes reinforces the identification of the tetragonal phase of SnO. Also, the well-defined, narrow, and intense Raman peaks due to the structural periodicity and symmetry of the crystal lattice confirm the material crystallinity.^29^ However, it is important to note that unlike XRD, the Raman technique does not reveal the vibrational modes associated with SnO_2_. Furthermore, when the Ni-doped SnO sample is examined, Raman spectroscopy results show no evidence of secondary phases related to nickel compounds such as NiO. Also, a reduction in the intensity of the *E*_g_ peak is observed. The decrease in intensity is associated with short-range disorder in the structure caused by the presence of Ni that polarizes the crystal lattice.^30^ The absence of Raman-active modes corresponding to nickel-based secondary phases together with the XRD data indicates that Ni is effectively incorporated into the SnO matrix.

As was mentioned, nanoparticle morphology control can improve adsorptive parameters and, consequently, enhance the material response. Figure 3A,B shows the SEM images of the pure and Ni-doped SnO films. It can be observed that both films are characterized by micrometric plates with a morphology trending toward the formation of square/rectangular microplates.

**Figure 3:**
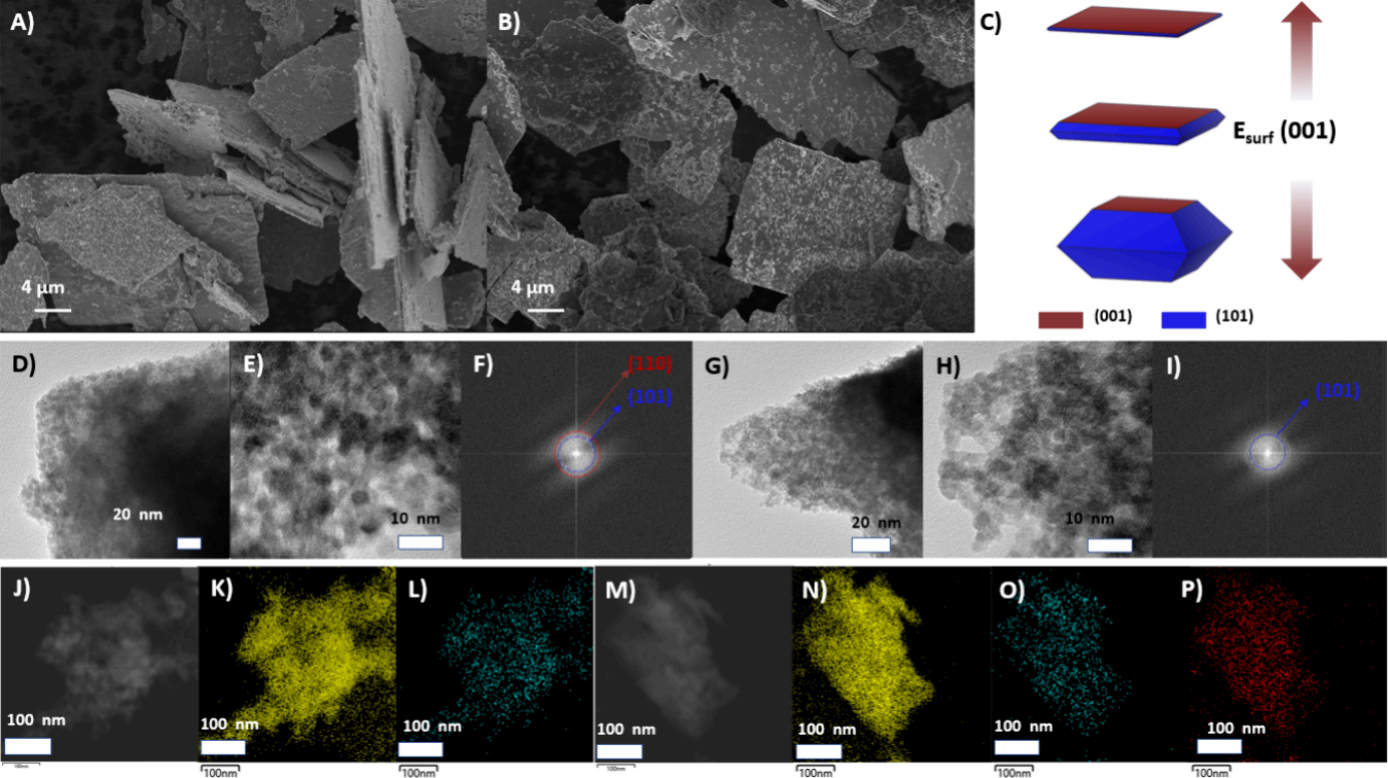
SEM images of the (A) SnO and (B) SnO:Ni samples. TEM images of the (C–E) SnO and (F–H) SnO:Ni samples. EDX mapping of the (I–K) SnO and (L–O) SnO:Ni samples.

This result indicates that aggregation between the microplates does not occur, which could have led to their stacking along the *c*-axis.^9^ Small particles can be observed on the plate surfaces, suggesting that they may be precursors in the formation of the microplates, characterized by a consistent yet incomplete self-assembly process.^31^ For the pure SnO film, the average plate lengths and thicknesses were 19.9 and 0.49 μm, respectively, while the Ni-doped sample exhibited average plate lengths and thicknesses of 22.4 and 1.09 μm, respectively. This result suggests that the short- and long-range alterations induced by Ni doping promote the growth of microplate thickness.

According to the Wulff construction, as described by Jaskaniec et al.,^32^ SnO microplates are predominantly formed by the (001) surface, while the (101) surfaces form the sides. Here, the nanoparticle morphologies (Figure 4A,B) were constructed considering the lowest-energy (001) and (101) surfaces (*E*_(001)_/*E*_(101)_ < 0.5), consistent with experimental stability results.^33^ When the (001) surface energy (*E*_surf_(001)) decreases with respect to (101) surface energy (*E*_surf_(101)), an increase in surface area (001) is expected, and a microplate morphology is observed (Figure 4A). Meanwhile, when doping with Ni, *E*_surf_(001) ≫ *E*_surf_(101), evolving into the morphology of a truncated octahedron (Figure 4B). Therefore, doping with Ni increases *E*_surf_(001), which leads to an increase in microplate thickness, as observed in Figure 3B.

**Figure 4:**
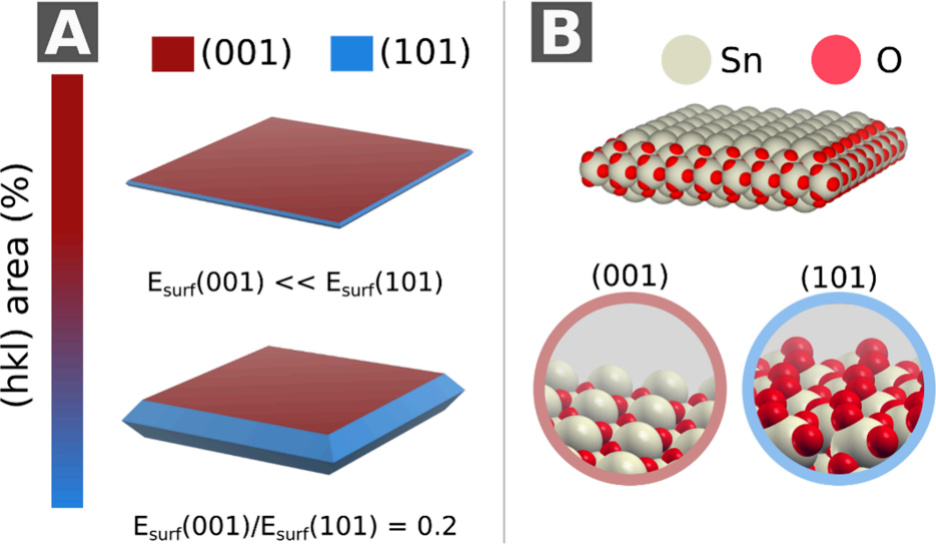
Equilibrium particle morphology of SnO predicted by the Wulff construction. (A) Evolution of particle shape with decreasing *E*(001)/*E*(101) energy ratio (<0.5), showing increased (001) facet exposure and progressive flattening into a quasi-2D structure. The insets show atomic structures of (001) and (101) surfaces (Sn = white, O = red) and the final platelet-like particle model (B) highlighting the dominant (001) basal planes.

TEM analyses were conducted along with EDX mapping to investigate the samples. It can be observed that the microplates in both samples consist of nanoparticles of approximately 6 nm (Figure 3C,D and 3F,G). The visualization of these particles solidifies the incomplete self-assembly process described earlier. The morphology of the SnO nanoparticles consists of truncated octahedron structures, particularly highlighting the (101) and (110) planes with interplanar distances of 2.99 and 2.68 Å, respectively (Figure 2E,H). The 2.68 Å interplanar distance can also be associated with the (101) plane of the SnO_2_ tetragonal phase, exclusively observed in the undoped sample. EDX mapping images were obtained to observe the atomic distribution of elements within the sample (Figure 3I–O). For the SnO sample, only the presence of O and Sn is observed, which are uniformly distributed throughout the sample. A similar result is observed for the SnO:Ni sample, with the presence of Ni also uniformly distributed throughout the sample. This result indicates that Ni was indeed incorporated throughout the sample without segregation.

Since the semiconductor-gas interface governs gas sensing, an in-depth analysis of the sample surfaces is mandatory. For this analysis, XPS analysis of both samples was carried out (Figure 5). As expected, from the survey spectra of the samples (Figure 5A), it was possible to observe that the SnO sample exhibits only signals related to Sn and O, whereas in the Ni-doped sample, an additional Ni signal is detected (component centered at 855.6 eV). The Sn 3d region was analyzed by using high-resolution spectra, as shown in Figure 5B.

**Figure 5:**
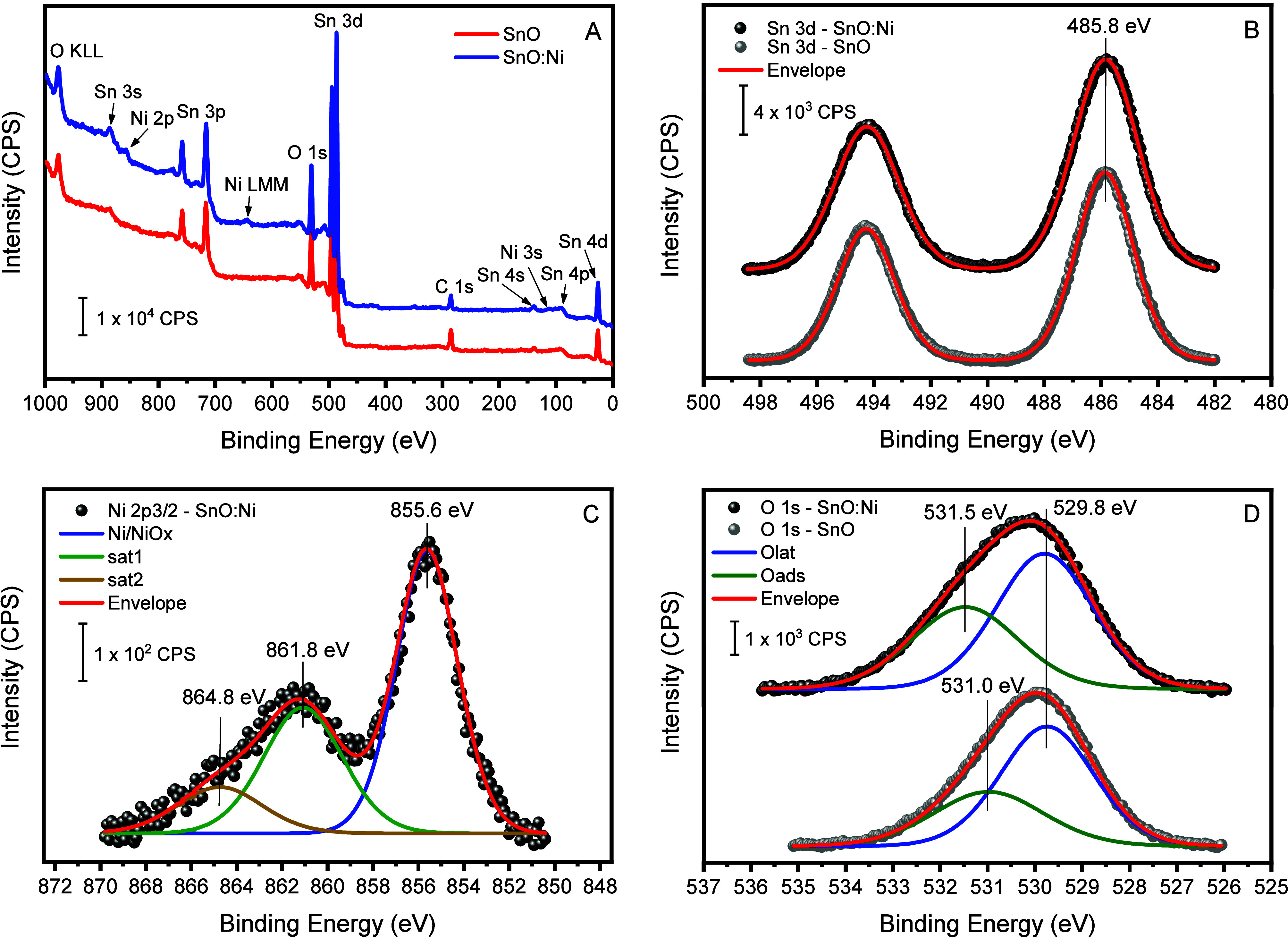
(A) Survey spectra of the samples SnO (pure, red line) and SnO (1% Ni, blue line). The labels point out the corresponding transitions for adventitious carbon, tin, oxygen, and nickel as indicated. High-resolution spectra B, C, and D of Sn 3d, Ni 2p_3**/**2_, and O 1s, respectively, for the samples SnO and SnO:Ni.

For both samples, this region of the spectrum was fitted using a single doublet corresponding to the spin–orbit coupling of Sn 3d_5/2_ and Sn 3d_3/2_ centered at 485.8 and 494.4 eV, respectively. Like the undoped sample, the Ni-doped sample exhibited only characteristic signals related to Sn^2+^, i.e., no signals were found on the surface of Sn^4+^.^34^ Contrary to what was reported for the case of nickel-doped SnO_2_, no shift of the Sn signal was observed that could be attributed to the substitution of Ni in the oxide lattice.

In Figure 5C, the spectrum region related to Ni 2p_3/2_ is presented. For the pure SnO samples, nothing was detected in this region, while signals appeared for the Ni-doped SnO sample. As it is well-known, the Ni photoelectron spectrum, among other transition metals, contains contributions from multiplet splittings, shakeup, and plasmon loss structures that make it difficult to accurately identify and quantify the different Ni species and/or oxidation states.^34,35^ Additionally, the low amount of Ni in the sample (1%) made this more complicated due to the poor signal-to-noise ratio. Nevertheless, a conservative analysis of the obtained Ni photoelectron spectrum shows that it can be fitted into a single component centered at 855.6 eV for a Ni 2p_3/2_ and two additional peaks at 861.8 and 864.8 eV, accounting for shakeup structures. This result is in agreement with the expected Ni^2+^ oxide state, even when the presence of Ni^3+^ species, e.g., as oxyhydroxide, could not be ruled out.^35–37^ The analysis of the O 1s region of the samples (Figure 5D) revealed that for both samples, there is more than one oxygen species, as evidenced by the shoulder on the high binding energy side. A two-peak model decomposition yields a low binding energy peak centered at 529.8 associated with lattice oxygen, O_lat_, from the SnO structure, and the high binding energy peak centered at 531.5 eV, which has been associated with oxygen ions such as O^2–^ in the oxygen-deficient regions^38^ and/or oxygen adsorbed species, such as hydroxyls or weakly bound surface oxygen, as expected for samples exposed to ambient conditions.^39^ Table 2 summarizes the surface atomic concentrations derived from the XPS analysis. The value obtained for the atomic ratio O_lat_/(Ni + Sn), together with the XRD and Raman results, allows us to assume that all Ni atoms are substitutional in the SnO lattice, occupying Sn sites and coordinating with surrounding O^2–^ anions within the SnO crystal structure.

**Table 2:** Surface Atomic Concentration (%) Obtained from XPS Spectra

	surface atomic concentration			atomic ratio	
	Sn	Ni	O_latt_	O_latt_/Sn	O_latt_/(Ni + Sn)
SnO	23.9		28.3	1.0	
SnO:Ni	27.2	0.9	28.3		1.1

The adsorption properties of pure and Ni-doped samples were studied by a diffuse reflectance spectroscopy (DRS). The band gap energy (*E*_g_) was calculated using the Tauc plot method and the Kubelka–Munk function,^19^ in which the *y*-axis is the transformed Kubelka–Munk function and the *x*-axis is the photon energy. The bandgap energies correspond to the crossing point between the extrapolation of the linear portion of the curves and the *x*-axis. The SnO direct band gap microplates are shown in Figure 6.

**Figure 6:**
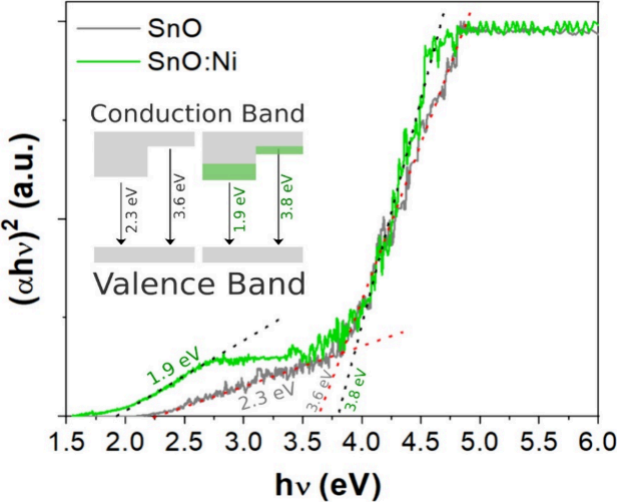
Reflectance spectra of the direct band gaps of SnO and SnO:Ni doped samples.

Two regions of interest are distinguished in Figure 6; the first zone between 1.5 and 2.5 eV corresponds to the direct transition between the valence and conduction band of tin monoxide SnO formed by the levels Sn-5s, Sn-5p, and O-2p.^40^ By extrapolating the linear portion of the Tauc plot in the optical absorption region, the estimated *E*_g_ values are 2.3 and 1.9 eV for SnO and SnO-Ni doped films, respectively. These values are lower than earlier reported values in the literature.^11,41,42^ It was found that the crystal quality of SnO increases and the optical band gap decreases.^11^ Li et al.^9^ suggest that after Ni being doped, the band gap energy is narrowed because of the *sp-d* interactions, the band electrons, and the localized *d* electrons of the Ni substituting Sn. In the band structure of SnO, the main contributions near the valence band maximum (VBM) come from the Sn 5*s* and the O 2*p* orbitals. In the conduction band, the O 2*p* component is relatively minor, while the states near the conduction band minimum (CBM) are primarily composed of Sn 5*p* orbitals. The *s-d* and *p-d* exchange interactions introduce a positive shift in the valence band edge and a negative shift in the conduction band edge, resulting in a narrowing of the band gap.

In the region of 3.5 to 4.0 eV, the values of *E*_g_ for undoped and Ni-doped samples are 3.6 and 3.8 eV, respectively, which are characteristic of SnO_2_ band gap values. It is well established that SnO_2_ is a wide-band gap (3.6 eV) semiconductor of n-type due to oxygen deficiency,^43^ and higher values can be attributed to the method of preparation, particle size, and nanofilm morphology. These results are in accordance with the X-ray diffraction technique, which suggests that SnO_2_ is inside the synthesized material and not on the surface.

### 3.2 Electrical Characterization

In semiconductor oxide gas detection, the interaction between the target gas and the metal oxide surface induces alterations in the material’s electrical conductivity, subsequently linked to gas concentration.^44^ Crucial for the gas detection mechanism are the surface reactions and desorption processes occurring on the metal oxide surface. One important factor that influences the sensor’s properties is the operating temperature since it greatly affects the adsorption species and the reaction rate of the gas. Also, the response process will change as the temperature fluctuates.^45^ The evolution of the conductance of the SnO and Ni-doped SnO samples as a function of time was studied in the operation conditions of air and 100 mmHg of CO at 250 °C for 40 h in order to investigate the stability of the semiconductor films. Figure S.M.1 shows that no significant changes in conductivity occurred over the period analyzed. The changes observed in conductivity are less than 1%. The experimental study indicates that temperature fluctuations in the surrounding atmosphere have little effect on the film conductivity. Variation of the ambient temperature causes fluctuations in the operating temperature, which can be accompanied by small changes in both the concentration of the charge carriers within the grains of the oxide semiconductor and the properties of the intergrain contacts.^45^

Moreover, metal oxide film resistance (*R*) often exhibits temperature-dependent behavior that can be consistently observed. This characterization aids in identifying the ideal temperature range for optimizing the gas detection performance. The Arrhenius equation, which adopts the form *G* = *Ae* ^(−*E*_a_/*kT*)^, which is used to describe the temperature dependency of the semiconductor oxide response, where *G* is the conductivity, *E*_a_ is the activation energy, and *k* is the Boltzmann constant.

By measuring the film resistance at different temperatures (*T*), it is possible to determine the *E*_a_ and the pre-exponential factor (*A*) of this activated process. These parameters can then be used to understand and optimize the semiconductor performance, including its response time. The Arrhenius equation can describe certain temperature-dependent phenomena in semiconductors, including the temperature dependence of carrier concentrations.^46^ Specifically, it can be used to describe the temperature dependence of the carrier concentration in semiconductors. This value is specific to the particular semiconductor oxide configuration and is typically determined experimentally. Also, it is important to observe that the pre-exponential factor (*A*) can vary depending on the specific gas being adsorbed on the surface, as different gases may have different affinities for the metal oxide surface and different reaction or desorption kinetics.^47^ The value of *E*_a_ is related to the mobility of the carriers to migrate from one grain to an adjacent one if there is band bending.^48^ A decrease in *E*_a_ indicates that electrons can easily move to neighboring grains. The temperature dependence of conductivity (*G* = 1/*R*) is shown in Figure 7.

**Figure 7:**
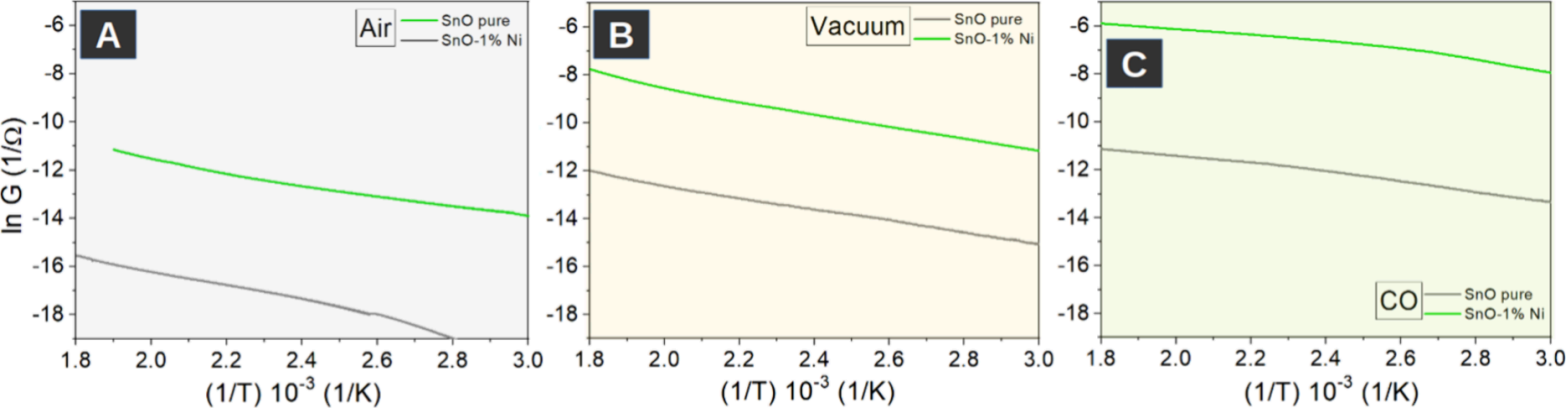
Arrhenius plots of thick films for undoped and doped-SnO under different atmospheric conditions: (A) air, (B) vacuum, and (C) carbon monoxide atmospheres.

It is possible to observe the variation of conductance as a function of inverse temperature for the samples in air, vacuum, and CO in order to analyze the performance of the undoped and doped samples in an oxidizing, neutral, and reductive atmospheres, respectively. Table 3 summarizes the *A* factor and the *E*_a_ obtained using eq 1 for the three different atmospheric conditions.

**Table 3:** *A* Factor and *E*_a_ Values Calculated from Arrhenius Plots

atmosphere	sample	*A*	*E*_*a*_ (eV)
air	SnO	2.39 × 10^–4^	0.33
	SnO:Ni	7.05 × 10^–3^	0.24
vacuum	SnO	3.35 × 10^–4^	0.21
	SnO:Ni	3.96 × 10^–2^	0.22
CO	SnO	5.52 × 10^–4^	0.17
	SnO:Ni	7.42 × 10^–2^	0.16

From Table 3, under air, vacuum, and CO, an increase in the *A* factor for the Ni-doped samples can be observed, especially under vacuum and CO atmospheres. Besides, for the same sample, the *A* factor increases when the material is exposed to the following sequence of atmospheres: air, vacuum, and CO. Specifically, for the Ni-doped sample, the increase in *A* is more pronounced, showing a higher carrier concentration. For a high oxygen pressure, it is expected that the surface has adsorbed ionized acceptors, which reduce the concentration of oxygen vacancies and electrons, which reflects a lower conductivity. In a vacuum or CO atmosphere, the concentrations of oxygen vacancies and electrons increase, which is consistent with the increase of *A* and the conductivity.^49,50^ Additionally, it can be seen that at any temperature and atmosphere, the conduction for the Ni-doped SnO sample is higher than that of the pure SnO. The conductivity is determined by the mobility and the density of carriers.^51^ As a general trend, an increase of the *A* factor favors the electrical conductance as a consequence of a higher concentration of charge carriers, which are mainly due to oxygen vacancies in the material. Also, it is in agreement with the slightest band gap found in the doping samples with nickel. Zakaria et al.^52^ also suggest that a larger grain size improves the electrical conduction, which could be related to the higher size of the Ni-doped sample.

To determine the working temperature (the temperature in which the sensitivity to CO is the highest) of the undoped and doped SnO films, we monitored their sensitivity to CO as a function of temperature, as shown in Figure 7C. The electrical conductivity as a function of the time of the samples was performed, as shown in Figure 8. The analysis of the sample’s responses revealed that the electrical conductivity (*G* = 1/*R*) of both samples decreased after exposure to air and increased after exposure to CO. This result is intriguing as it pertains to an n-type semiconductor behavior,^53–55^ while SnO is often described in the literature as a p-type semiconductor, primarily due to the presence of tin vacancies.^56–58^ According to Suman et al.,^54^ SnO with direct bandgap values greater than 2.5 eV can exhibit either n-type or p-type semiconductor behaviors. The type of semiconductor is determined by the doping employed or its redox level. Since there are no observed changes in the semiconductor type with Ni doping, we can infer that this behavior is directly linked to the method of synthesis used. As the synthetic environment has low O_2_ concentration due to being a closed system, coupled with the high-temperature urea hydrolysis,^53^ it creates a reducing environment, causing the synthesized SnO samples to have oxygen deficiencies in their structure, consequently exhibiting an n-type behavior. This behavior is consistent with what has been observed in previous studies.^59–61^

**Figure 8:**
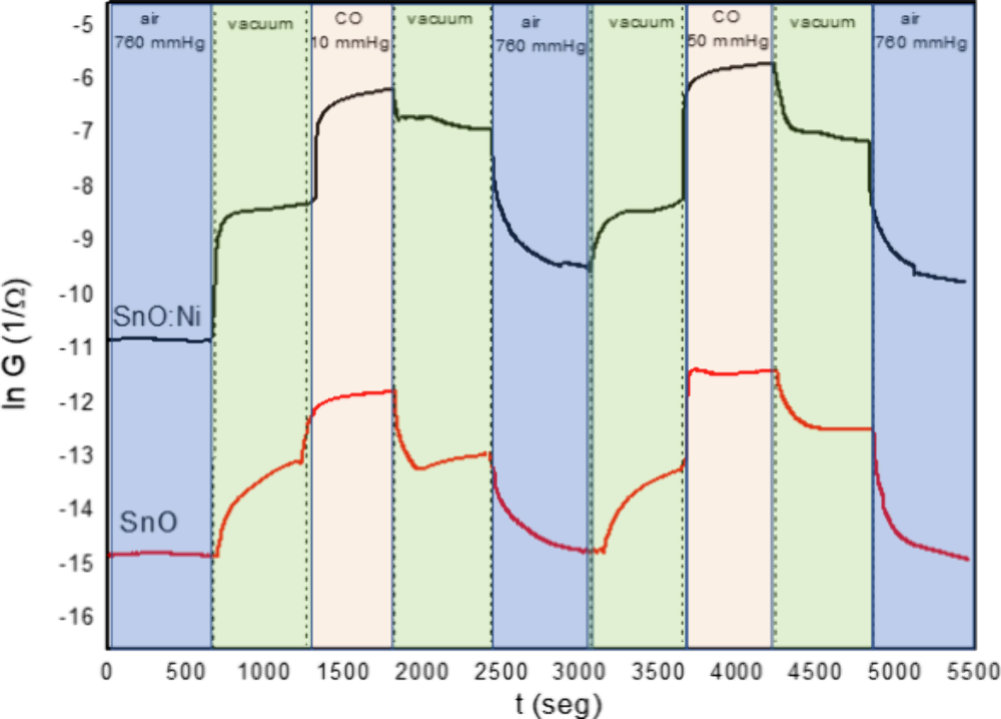
Electrical conductance as a function of time and atmosphere for the samples at 250 °C.

Figure 8 also allows for calculating the recovery time (*t*_rec_) for both samples in the presence of 10 and 50 mmHg CO. Time recovery in gas sensors refers to the ability of the sensor to return to its baseline state after exposure to a gas. The SnO sample exposed to 10 and 50 mmHg CO presented *t*_rec_s of 60 and 179 s, respectively. Furthermore, the Ni-doped SnO sample exposed to 10 and 50 mmHg CO presented *t*_rec_s of 27 and 99 s, respectively. Masteghin and Orlandi^62^ also obtained SnO nanostructures that reached a sensor response time of 60 s using an operating temperature of 100 °C and CO concentrations similar to those used in this work. Also, Suman et al.^61^ reported a sensor signal (*R*_CO_/*R*_air_) of 0.8 for SnO nanobelts at 200 °C for CO. The sensor signal calculated for the doped sample of SnO:Ni in this work at 200 °C for CO is 0.52. The inclusion of Ni within SnO films clearly enhances CO sensing performance when compared to pure NiO. For instance, Simion et al.^63^ reported that NiO-based sensors operated at 250 °C exhibited a response time of 9 min for 50 ppm CO under 50% RH conditions. Likewise, another study by Khaleed et al.^64^ also found relatively long response times for CO detection using undoped NiO sensors. In contrast, our Ni-doped SnO microplates demonstrate significantly faster kinetics: the response time under similar CO concentration and temperature conditions is approximately 2–3 orders of magnitude shorter than those reported for pure NiO. This improvement can be attributed to the synergistic effect of Ni doping in SnO, which promotes enhanced electrical conductivity and more active surface interactions with CO molecules. Therefore, it could be demonstrated that Ni-doping can meaningfully improve the SnO sensing properties.

Finally, the CO reaction mechanism was previously proved by Mirabella et al. and can be expressed as follows.^65^

$$A+{O^{0}}_{x}\to \mathrm{AO}+V^{2+}+2e$$ 1

where A correspond to a reductive gas such as CO, O^0^_*x*_ refers to the lattice oxygen, V^2+^ refers to the double ionized vacancy formation and e denotes the electrons. The electrons, released during the formation of oxygen vacancies, increase the number of available carriers at the conduction band, promoting an increase in the conductivity of doped and undoped films when reacting with CO.

### 3.3 Structural Models

Periodic DFT models were employed to simulate bulk SnO and Ni-doped SnO (001) surfaces using a 3 × 3 × 3 supercell (72 SnO units) and a 3 × 3 slab (54 SnO units) for bulk and surface, respectively. The slab model consisted of four lamellas, each with two Sn atomic layers (Figure 9A–B) separated by a 15 Å vacuum layer to avoid periodic interactions. Ni doping was introduced via single Sn substitutions (<2% concentration) at both the outermost Sn layer (Figure 9C) and the second Sn layer (Figure 9D) to probe depth-dependent effects, with full relaxation of atomic positions and lattice parameters to evaluate doping-induced strain. For CO adsorption, the molecule was positioned at valley regions (Figure 9C–D) and top surface atoms (Sn/Ni/O), testing both C- and O-anchoring configurations (Figure 10), followed by structural relaxation to identify the most stable geometries.

**Figure 9:**
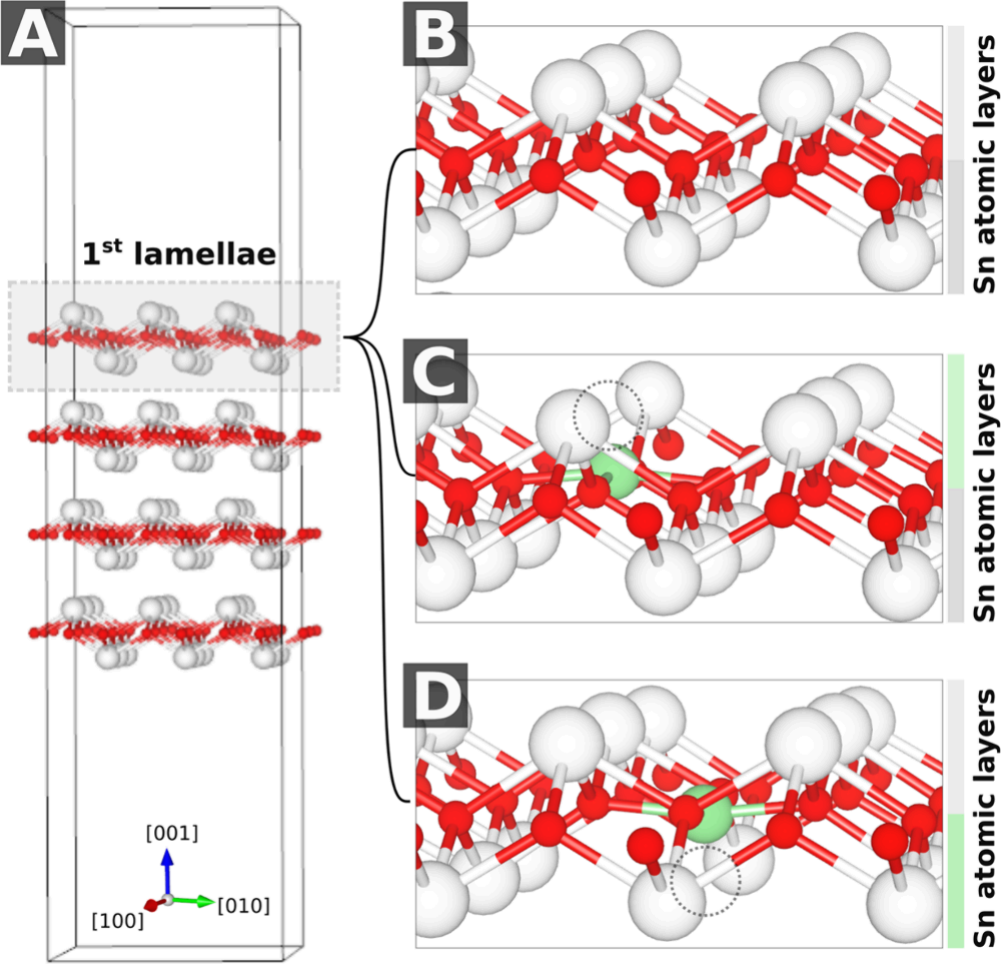
(A) 3 × 3 supercell of the four-lamella (001)-SnO surface, where each lamella consists of two Sn (white) atomic layers. (B) Outermost lamella of pristine SnO. (C) Ni substitution (green) at the external Sn site and (D) at the second Sn layer. All structures are shown postrelaxation; dotted circles in (C) and (D) mark the initial Ni positions prior to optimization.

**Figure 10:**
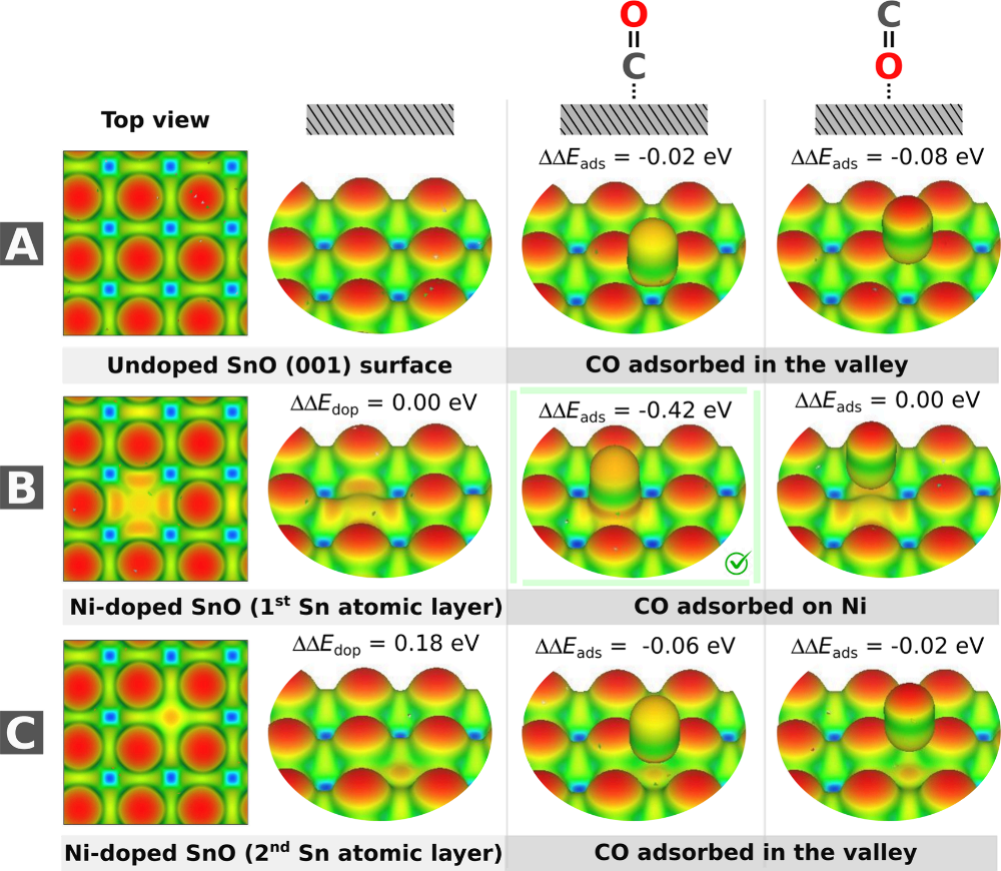
Charge density difference analysis for (A) pristine SnO (001), (B) Ni-doped SnO (outermost Sn layer), and (C) Ni-doped SnO (second Sn layer). The left panels show the cleaned surfaces, while the right panels show the most stable CO adsorption configurations (C-bonded or O-bonded) for each system. The isosurfaces show the total charge density ρ­(r) at 0.01 |e|·bohr^–^^3^, colored by the charge density difference ρ­(*r*) – ∑ρ_atoms_(*r*). The blue and red colors indicate electron depletion and accumulation, respectively. Ni dopants induce localized charge reorganization, with stronger charge density difference modulation for surface doping (B) than for the subsurface (C).

Structural relaxations converged to 10^–6^ Ry in energy and 10^–3^ Ry/Bohr in forces, ensuring accurate geometries for subsequent analysis (Figures 8 and 9).

The adsorption energy (*E*_ads_) of CO on Ni-doped SnO (Figure 2) was calculated as *E*_ads_ = *E*_surface+CO_ – *E*_surface_ – *E*_CO_, where *E*_surface+CO_, *E*_surface_, and *E*_CO_ are the total energies of the adsorbed system, the clean surface, and the isolated CO molecule, respectively.

Charge density difference isosurfaces (Figure 10) were computed to visualize electron redistribution: Δρ = ρ_surface+CO_ – ρ_surface_ – ρ_CO_, plotted at ± 0.01 |*e*|·Bohr^–3^ (blue/yellow for accumulation/depletion). Lewis acidity/basicity sites were identified via electrostatic potential maps and charge analysis.^33,66^

### 3.4 Computational Evidence of CO Adsorbed on the (001) Surface

The SnO 3 × 3 × 3 supercell was fully optimized at the PBE-D3 level and revealed a minor but systematic volume contraction upon Ni doping (<2%), with the unit cell volume decreasing from 1905.28 Å^3^ (pristine) to 1901.88 Å^3^ (doped). This reduction stems from the significantly shorter Ni–O bonds (1.88 Å, planar coordination) compared to Sn–O bonds (2.25 Å), as evidenced by the atomic coordinates (Figure 9B,C) and ionic radius (Ni^2^^+^ = 0.69 Å vs Sn^2^^+^ = 1.18 Å) difference. The equal lengths of all four Ni–O bonds (1.88 Å) confirm the dopant’s local structural symmetry (∼*D4h*), while the Sn–O polyhedra exhibit a well-known nonplanar structure due to Sn’s active lone pair. The anisotropic contraction (*a*, *b* axes shrink by 0.15%, *c* expands by 0.13%) suggests that Ni doping preferentially affects in-plane interactions, consistent with the layered SnO structure (Figure 9A).

The four-lamella SnO (001) slab, with each lamella comprising two Sn atomic layers (Figure 9A), exhibited a pronounced Ni doping preference for surface sites, as evidenced by depth-dependent substitution energies: 0.00 eV for the outermost Sn layer, +0.18 eV for the second layer, and +0.18–0.20 eV for deeper layers. This energetic gradient reflects the combined effects of reduced coordination at surface sites and strain relief from shorter Ni–O bonds. The nearly identical substitution energies for the third and fourth layers indicate minimal thermodynamic driving force for Ni incorporation beyond the second atomic layer under equilibrium conditions. The four-lamella SnO (001) slab maintained its characteristic lamellar structure after Ni doping, with each lamella (comprising two Sn atomic layers; Figure 9A) preserving the (001) surface topology despite local coordination changes.

The local structural perturbations induced by Ni doping were observed throughout the electronic changes. However, the lamellar (001) surface architecture ensured that charge density differences remained confined to doped regions (Figure 10). Notably, second-layer doping produced no measurable electronic perturbation in the first lamella where CO adsorption occurs, evidenced by nearly identical adsorption energies between pristine (−0.02 eV for C-binding, −0.08 eV for O-binding) and second-layer doped systems (−0.06 eV for C, −0.02 eV for O). First-layer Ni doping, however, dramatically enhanced CO affinity through carbon coordination (−0.42 eV vs pristine), while oxygen-bound configurations became unstable (0.00 eV). This site-specific behavior confirms that the Ni electronic influence is highly localized, as deeper dopants (third/fourth layers: −0.03 to −0.09 eV) showed negligible effects versus pristine SnO. The preservation of adsorption energetics beyond the first doped lamella underscores the material’s ability to maintain intrinsic surface properties while achieving targeted reactivity modifications at selected atomic layers.

The charge density difference map, shown in Figure 10B, reveals how Ni doping modifies the electronic structure of the SnO surface. The plot shows significant electron redistribution localized near the dopant site, visible as a red region (electron accumulation) surrounded by characteristic yellow/light green square-shaped zones (electron depletion). This pattern demonstrates charge transfer toward the Ni center, confirming the dopant’s strong local influence on electron distribution. Notably, the charge reorganization is more pronounced for surface doping (Figure 10B) than for subsurface doping (Figure 10C), with electron accumulation primarily concentrated within the immediate coordination sphere of the Ni atom. In short, the spatial confinement of these electronic changes to the dopant vicinity explains why the modified surface properties remain localized without significantly affecting neighboring lamellae.

## 4 Conclusions

Undoped and Ni-doped tin monoxide (SnO) samples were synthesized using a microwave-assisted hydrothermal method to evaluate their structural and electrical properties for carbon monoxide (CO) detection. Both undoped and doped materials primarily formed SnO microplates, with a small amount of SnO_2_ present in the bulk, particularly in the Ni-doped sample. XRD analysis showed that pure SnO was dominated by the (001) plane, while the doped sample exhibited both (001) and (101) planes. Interestingly, XRD and DRS suggested that SnO_2_ existed within the material. However, Raman spectroscopy and XPS detected only Sn^2+^ species on the surface of both materials. Electrically, the Ni-doped sample showed decreased activation energy (*E*_a_) in the air. This translates to improved conductivity. No significant *E*_a_ changes were observed in a vacuum or carbon monoxide for either sample. Notably, Ni doping significantly increased carrier concentrations and reduced the band gap energy, leading to higher conductivity. This, combined with the lower *E*_a_ in air, makes Ni-doped SnO an excellent candidate for room-temperature CO sensors, which is highly desirable. Finally, density functional theory (DFT) simulations indicated that first-layer Ni doping strongly enhanced the CO affinity through carbon coordination, making oxygen-bound configurations unstable.

## Supplementary Material



## Data Availability

The raw/processed data required to reproduce these findings cannot be shared at this time due to technical or time limitations.

## Notes

Time response of the conductivity (DOCX)
