# Supplementary material for: Ni-Doped SnO Microplates for Carbon Monoxide Gas Detection
Source: ACS Omega. 2025 Oct 7;10(41):48603–13. doi: 10.1021/acsomega.5c06392 (PMC12547786; doi:10.1021/acsomega.5c06392)
Supplement: Supplementary file 1 [file ao5c06392_si_001.docx]

**SUPLEMENTARTY MANUSCRIPT**

**Ni-doped SnO microplates for carbon monoxide gas detection**

Giuliana Giulietti^a^, Miguel D. Sanchez^b^, Elson Longo^c^, Marcelo Assis^d^, Anderson Albuquerque^e^, Julio R. Sambrano^f *^, Miguel A. Ponce^f-g^, Paula M. Desimone^a^

^a^ Institute for Research in Materials Science and Technology (INTEMA), National University of Mar del Plata (UNMdP), Mar del Plata, Argentina.

^b^ Instituto de Física del Sur (IFISUR), Departamento de Física, Universidad Nacional del Sur (UNS), CONICET, Bahía Blanca, Argentina

^c^ Center for Research and Development of Functional Materials (CDMF), Federal University of São Carlos (UFSCar), São Carlos, SP, Brazil.

^d^ Department of Biosciences, Federal University of São Paulo (UNIFESP), Santos, Brazil

^e^ Instituto de Química, Universidade Federal do Rio Grande do Norte, UFRN, Natal, RN, Brazil

^f^ Modeling and Molecular Simulations Group, São Paulo State University (UNESP), School of Sciences, Bauru, SP, Brazil.

^g^ CIFICEN (UNCPBA-CICPBA-CONICET) and Instituto de Física de Materiales Tandil (UNCPBA), Tandil, Argentina

**Figure S.M.1**
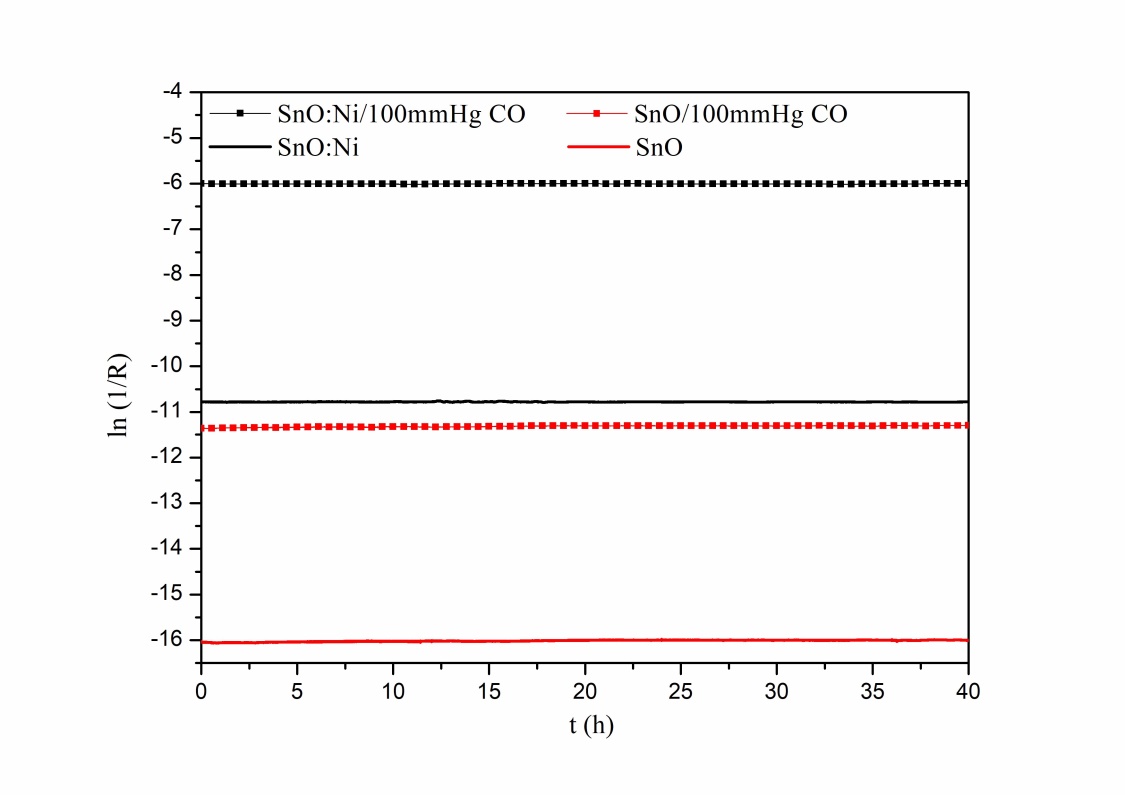


**Figure S.M.1.** Time response of the Conductivity. No significant changes in conductivity occur over the period analyzed of 40h
